# A Comparison of Computational Methods for Identifying Virulence Factors

**DOI:** 10.1371/journal.pone.0042517

**Published:** 2012-08-03

**Authors:** Lu-Lu Zheng, Yi-Xue Li, Juan Ding, Xiao-Kui Guo, Kai-Yan Feng, Ya-Jun Wang, Le-Le Hu, Yu-Dong Cai, Pei Hao, Kuo-Chen Chou

**Affiliations:** 1 Hubei Bioinformatics and Molecular Imaging Key Laboratory, Huazhong University of Science and Technology, Wuhan, Hubei, China; 2 Shanghai Center for Bioinformation Technology, Shanghai, China; 3 Institute of Systems Biology, Shanghai University, Shanghai, China; 4 Department of Chemistry, College of Sciences, Shanghai University, Shanghai, China; 5 Key Laboratory of Systems Biology, Shanghai Institutes for Biological Sciences, Chinese Academy of Sciences, Shanghai, China; 6 Pathogen Diagnostic Center, Institute Pasteur of Shanghai, Chinese Academy of Sciences, Shanghai, China; 7 Shanghai Center for Systems Biomedicine, Shanghai Jiaotong University, Shanghai, China; 8 Department of Medical Microbiology and Parasitology, Institutes of Medical Sciences, Shanghai Jiao Tong University School of Medicine, Shanghai, China; 9 Gordon Life Science Institute, San Diego, California, United States of America; 10 Schepens Eye Research Institute, Harvard Medical School, Boston, Massachusetts, United States of America; Hospital for Sick Children, Canada

## Abstract

Bacterial pathogens continue to threaten public health worldwide today. Identification of bacterial virulence factors can help to find novel drug/vaccine targets against pathogenicity. It can also help to reveal the mechanisms of the related diseases at the molecular level. With the explosive growth in protein sequences generated in the postgenomic age, it is highly desired to develop computational methods for rapidly and effectively identifying virulence factors according to their sequence information alone. In this study, based on the protein-protein interaction networks from the STRING database, a novel network-based method was proposed for identifying the virulence factors in the proteomes of UPEC 536, UPEC CFT073, *P. aeruginosa* PAO1, *L. pneumophila* Philadelphia 1, *C. jejuni* NCTC 11168 and *M. tuberculosis* H37Rv. Evaluated on the same benchmark datasets derived from the aforementioned species, the identification accuracies achieved by the network-based method were around 0.9, significantly higher than those by the sequence-based methods such as BLAST, feature selection and VirulentPred. Further analysis showed that the functional associations such as the gene neighborhood and co-occurrence were the primary associations between these virulence factors in the STRING database. The high success rates indicate that the network-based method is quite promising. The novel approach holds high potential for identifying virulence factors in many other various organisms as well because it can be easily extended to identify the virulence factors in many other bacterial species, as long as the relevant significant statistical data are available for them.

## Introduction

The *Escherichia coli* O104:H4 bacteria outbreak since May-02-2011 in Germany has brought into focus the need to use reagents to rapidly identify pathogenic organisms and genes involved in the mechanisms of pathogenicity. Although the majority of *E. Coli* strains are beneficial to human bodies, the genome of this new strain of O104 was modified by mutations or the genetic materials secreted from other bacteria, rendering it able to produce Shiga toxin and resist to many kinds of antibiotics and also to the mineral tellurium dioxide, causing foodborne illness [1]. In the course of pathogens infection and pathopoiesis, virulence factors (VFs) play a key role. VFs are the molecules produced by pathogens that increase the ability of pathogens to cause disease. According to their mechanisms and functions, VFs can be generally classified into the following seven groups: (1) adhesins that attach microbes to their hosts, (2) colonization factors that enable certain bacteria to colonize within host cells, (3) effectors that suppress hosts’ defenses, (4) invasions that disrupt the host membranes and stimulate endocytosis, (5) toxins that poison the host cells and cause tissue damage, (6) capsular polysaccharides that protect pathogens from host defenses, and (7) siderophores that take up iron [2]–[4]. House-keeping proteins that are required for maintaining the basic cellular functions and are not related to pathogenesis are not virulence factors [2]. Therefore, virulence factors can be the potential targets of drugs to treat infectious diseases specifically, without killing or inhibiting other bacterial growth, avoiding the higher evolutionary pressure to develop drug resistance [4].

At present, complete genome sequences of almost all major bacterial pathogens have been determined (http://cmr.jcvi.org/tigr-scripts/CMR/CmrHomePage.cgi), providing significant insights into microbial pathogenesis and drug resistance. Meanwhile, several repositories aiming at collecting the virulence factors with their structures, functions and mechanisms have also emerged, facilitating the study of virulence factors of bacterial pathogens. The Virulence Factor Database (VFDB, http://www.mgc.ac.cn/VFs/), constructed by the virulence-guided classification system, currently contains 409 virulence factors and 2,353 VF-related genes (accessed June 2011) [5]. The Lawrence Livermore National Laboratory Virulence Database (MvirDB, http://predictioncenter.llnl.gov/) integrates DNA and protein sequence information from various databases and provides a browser tool enabling keyword and virulence classification search [6]. Researchers have identified many genes of potential virulence factors by analyzing comparative genomics or homology searching against the virulence factor databases. For example, Gulig et al. [7] identified 80 genes exclusively found in clade 2, which was the predominant clade among the clinical strains and generally possessed higher virulence potential in the animal models of *Vibrio vulnificus*. Conducting the investigation with a different approach, Wegmann et al. [8] used BLASTP to search against the toxin in the virulence factor database MvirDB to assess the GRAS (generally regarded as safe) status of *L. lactis* MG1363. Although the data relevant to virulence factors are expanding rapidly, it is still quite limited in the area of using computational tools to interpret, identify and characterize virulence factors. A large number of proteins in the microbial genomes are still annotated as hypothetical or with little functional characterization, or with contradictory information to confuse the comparative genomics analysis. Homology searching methods like BLAST [9] could only identify conserved virulence factors but failed to identify novel virulence factors that are evolutionary distant from known virulent proteins. In order to deal with such situation, several machine-learning approaches have been proposed, such as SPAAN [10] for identifying adhesins and adhesin-like proteins and VICMpred [11] for classifying bacterial proteins among the following four different functional classes: cellular process, information molecule, metabolism molecule and virulence factors. However, the former was restricted to adhesins only, while the latter was trained with merely 670 gram-negative bacterial proteins [10], [11]. To improve these kinds of situations, VirulentPred [12] and Virulent-GO [13] were developed recently for predicting bacterial virulent proteins based on their sequences information alone: the samples in the former were formulated by a vector consisting of five kinds of sequence features; while the samples in the latter by a vector containing the GO [14] information. It was reported that the two predictors yielded an overall success rate of 81.8% [12] and 82.5% [13], respectively.

The present study was devoted to develop a novel network-based method by incorporating the information of protein-protein interaction (PPI) for identifying bacterial virulence factors in UPEC 536, UPEC CFT073, *P. aeruginosa* PAO1, *L. pneumophila* Philadelphia 1, *C. jejuni* NCTC 11168 and *M. tuberculosis* H37Rv. Compared with the sequence-based methods such as BLAST, feature selection and VirulentPred, the network-based method achieved a remarkable improvement with the identification accuracy of 0.9. Further analysis showed that the functional associations such as the gene neighborhood and co-occurrence were the primary associations between these virulence factors in STRING database. The high success rates indicate that the network-based method is quite promising. It is anticipated that with the increasing amount of PPI networks available in more and more organisms, the current network-based approach will play a more and more important role in both applications and stimulating new strategies for in-depth investigation into the relevant areas.

## Materials and Methods

### 1. Benchmark Dataset

Datasets of virulence factors were downloaded from VFDB [5], a well-established database based on experimentally validated virulence factors extracted from literatures and supplemented with comprehensive genomics information from bacterial pathogens. A total of 2,295 proteins of virulence factors were obtained, involving 24 pathogens from *Bacillus* to *Yersinia*.

According to the total amount of virulence factors in each of these species, we selected five of them that contained the largest amounts of virulence factors. These five species were: (i) *Escherichia coli* 536 (UPEC 536), (ii) *Pseudomonas aeruginosa* PAO1 (*P. aeruginosa* PAO1), (iii) *Salmonella enterica (serovartyphimurium)* LT2, (iv) *Escherichia coli* CFT073 (UPEC CFT073), and (v) *Legionella pneumophila* Philadelphia 1 (*L. pneumophila* Philadelphia 1). The numbers of virulence factors in the above five species were 230, 190, 165, 117 and 117, respectively. Since these five species are closely related, we also selected another two species that were distant in phylogeny. The two species were *Campylobacter jejuni* NCTC 11168 (*C. jejuni* NCTC 11168) and *Mycobacterium tuberculosis* H37Rv (*M. tuberculosis* H37Rv), and they contained 98 and 86 virulence factors, respectively. All the aforementioned species, except *Salmonella enterica (serovartyphimurium)* LT2, were included in the STRING database [15]. Consequently, the virulence factors in the remaining six species would form our first-hand dataset.

The protein-protein interaction (PPI) network used here was retrieved from the STRING database [15] (http://string-db.org/). For each of the six species, a PPI network was constructed by integrating different sources of information derived from experimental, computational, and text-mining methods. Furthermore, all interactions in STRING are provided with a probabilistic confidence score, representing a rough estimate of how likely a given interaction describing a functional linkage between two proteins might occur. In order to predict virulence factors based on the STRING database, we extracted all the proteins and interactions between them for the 6 species mentioned above. Mapping these known virulence factors from VFDB to STRING proteins, we found 207, 110, 189, 116, 98 and 83 proteins for UPEC 536, UPEC CFT073, *P. aeruginosa* PAO1, *L. pneumophila* Philadelphia 1, *C. jejuni* NCTC 11168, and *M. tuberculosis* H37Rv, respectively, by BLASTP with the cutoff of HSP score being 90. These proteins comprised our positive dataset. Proteins, not known as virulence factors, were randomly selected from the remaining proteins of each species in STRING to compose the negative dataset, with the ratio between the size of negative dataset and positive dataset equaling 5∶1. Then, all the virulent and non-virulent sequences of the six species were randomly divided into a training dataset with a proportion of 80% and a testing dataset with 20%. The training dataset was used by the jackknife cross-validation method to assess the identification performance of each virulence factor classifier developed by us, while the testing dataset was used to compare our methods with other existing tools (such as VirulentPred) in identifying the virulence factors.

### 2. STRING Network-Based Method

It has been demonstrated that the STRING network-based method could be used to predict protein phenotypes [16]. The prediction accuracy thus obtained was 65.4% for budding yeast, much higher than the success rate (15.4%) by a random guess. In this study, we are to apply this novel method to predict virulence factors. In the PPI network, when predicting whether a protein was a virulence factor or not, we considered two kinds of information: the number of its neighbor nodes (proteins) and the strengths of its interactions (confidence scores) with them. The detailed process of the prediction based on STRING network is described as follows.

Firstly, suppose a PPI network consisting of n nodes 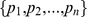, in which each node is divided into 2 classes (T = [T_1_, T_2_]), where T_1_ stands for “virulence factor”, T_2_ the “non-virulence factor”. Then we denoted the class of the *i*-th protein in the PPI network by


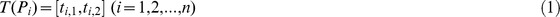


where


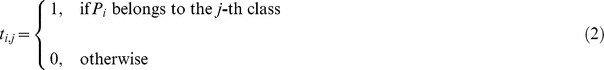


For a query protein 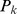, its interaction weights with m proteins (nodes) can thus be defined by


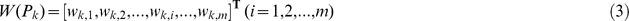


where 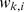 is the interaction weight (confidence score) between 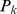 and the 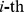 protein 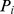 in the dataset concerned. If there is no interaction between them, let 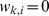. Since the self-interaction of proteins was not taken account here, we have 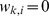 when 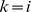. In order to estimate the likelihood of the protein 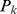 belonging to the 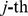 class, we defined a score function as given by


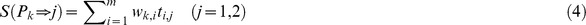


where proteins without any associations with the queried protein would have no contribution to the score function 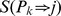. Thus, the likelihood of protein 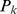 belonging to the 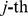 class can be deemed as the sum of the interaction weights of all its neighbor proteins being labeled as the 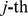 class in the training dataset. Apparently, the larger the value of 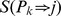, the more likely the protein 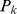 would belong to the 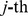 class. Thus, the class of the queried protein 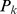 can be determined by the following formula:


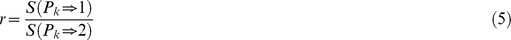


If 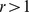, the queried protein 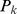 was predicted belonging to the virulence factors; otherwise, other kinds of proteins.

### 3. BLAST

For the purpose of comparison, we also used BLAST to predict the virulence factors as follows. First, let us denote the training set as 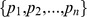, and a queried protein as 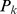, then comparing the queried protein 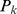 with the training set proteins by BLASTP with default parameters. In the list of hit results 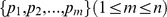, we chose the positive and negative samples both with the smallest e-values. If either positive or negative sample did not exist in the list, the corresponding e-value was set at 10. We computed the ratio of positive vs. negative samples’ e-values by the following equation:


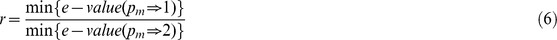


where 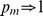 means the protein 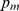 was a virulence factor; 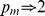, not. Obviously, the queried protein is more likely to belong to the same class as the hit protein with the smallest e-value in the hit list. Thus, if 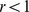, the queried protein 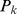 was assigned to the category of virulence factors; otherwise, other kinds of proteins.

### 4. Amino Acid and Pseudo Amino Acid Composition

In this method, virulence factors were coded by amino acid composition (AAC) and pseudo-amino acid composition (PseAAC) [17], from which some important features are selected by the feature selection method. Generally, the frequency of the occurrence of each amino acid in a protein sequence can be used to code the sequence. That is, a protein can be represented by a 20-D (dimensional) numerical vector. However, this traditional amino acid composition nearly loses the sequence-order information completely. To improve it, the pseudo amino acid composition (PseAAC) was proposed [17], [18] to complement the simple amino acid composition (AAC) for representing the sample of a protein. Since the concept of PseAAC was introduced, it has been widely used to study various problems in proteins and protein-related systems, such as predicting subcellular location of proteins [19], structural classes of proteins [20] and DNA-binding proteins [21], etc. In this study, we only employed the sequence-order information reflected by a series of PseAAC components [17] to code proteins. These kinds of sequence-order information were derived according to the following five physicochemical and biochemical properties of amino acids: (i) codon diversity, (ii) electrostatic charge, (iii) molecular volume, (iv) polarity, and (v) secondary structure propensity. The values of such five properties were retrieved from [22]–[24]. To get the optimal results, we set ë = 50 and ù = 0.15 for the PseAAC, as done in [24]. Since each of the aforementioned five features can generate ë = 50 discrete numbers, each protein sample will be coded by a (20+50×5 = 270)-D vector in the feature space.

**Table 1:** Prediction based on BLAST.

Species	*TP*	*FP*	*TN*	*FN*	*Sn*	*Sp*	*AC*	*MCC*
UPEC 536	74	105	694	87	0.45963	0.86859	0.80000	0.31484
UPEC CFT073	45	72	339	41	0.52326	0.82482	0.77264	0.31035
*L. pneumophila* Philadelphia 1	39	91	359	50	0.43820	0.79778	0.73840	0.20481
*P. aeruginosa* PAO1	21	24	159	17	0.55263	0.86885	0.81448	0.39494
*C. jejuni* NCTC 11168	35	69	311	40	0.46667	0.81842	0.76044	0.25190
*M. tuberculosis* H37Rv	34	52	262	32	0.51515	0.83439	0.77895	0.31646

### 5. Feature Selection and NNA Classifier

In machine learning, feature selection is a technique that selects an optimal subset of features to build a more robust learning model. Here, we used Maximum Relevance Minimum Redundancy (mRMR) method [25] to rank the 270 features based on their relevance to the classification variable (maximum relevance) and the redundancy among them (minimum redundancy). More important features will be selected earlier and ranked in higher position. Meanwhile, in spite of the features being ranked according to mRMR criteria, it is a bit of a challenge to get the optimal number of features used for the prediction. To solve the problem, we adopted Incremental Feature Selection (IFS) [26] to find the optimal number of features. For the 270 features ranking from higher to lower, we added features one by one to code the protein. Thus, we obtained a series of feature subsets


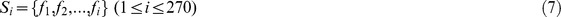


where 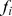 is the *i*-th feature in the ranked feature list. Subsequently, a Nearest Neighbor Algorithm (NNA) [27] classifier was constructed for each feature subset to predict whether a protein was a virulence factor or not. NNA is one of the simplest and most effective machine learning algorithms, which assigns the unknown sample to the class of its nearest neighbor. The core of this algorithm is the distance function:


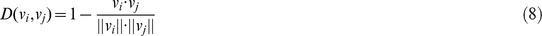


where 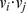 is the inner product of the two coding vectors 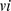 and 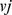, and 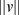 represents the module of vector v. Since each protein is coded by an i-D (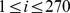) vector and the training set contains n proteins 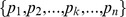, we can determine the class of a queried protein p as follows


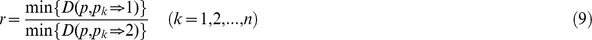


where 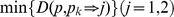 is the nearest distance of the queried protein p and the j-th class protein 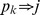, in which 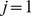 means protein 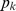 belongs to positive samples and 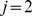 negative samples. According to the theory of NNA, if 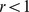, the queried protein is assigned to the virulence factors; otherwise not. Since the NNA classifier can be applied for every feature subset to perform a prediction, we draw an IFS (Incremental Feature Selection) curve to reflect the relationship between the performance of the NNA classifier and the feature subset. In the curve, x-axis is the number of features of the subset 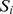 and y-axis is the prediction accuracy of the NNA classifier. The optimal prediction result is the highest point in the curve, which corresponds to the feature subset in the x-axis that achieves the highest overall accuracy in the curve.

**Figure 1:**
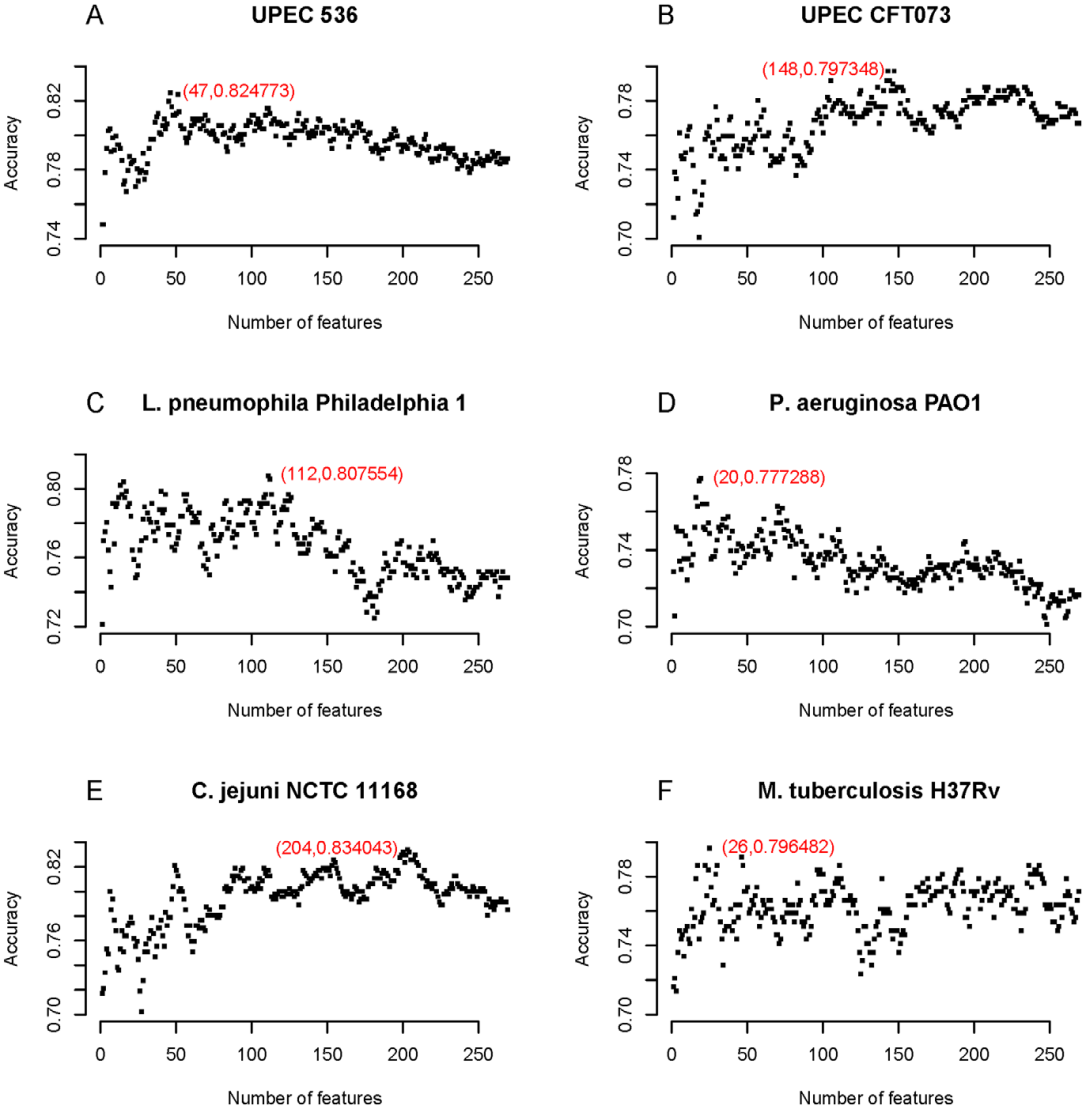
The IFS curve for each of the six species. It shows the relationship between the prediction accuracies of the NNA predictor and the number of feature subsets. The optimal feature subset is determined when the IFS curve arrives at the apogee. (A) UPEC 536; (B) UPEC CFT073; (C) *L. pneumophila* Philadelphia 1; (D) *P. aeruginosa* PAO1; (E) *C. jejuni* NCTC 11168; (F) *M. tuberculosis* H37Rv.

**Figure 2:**
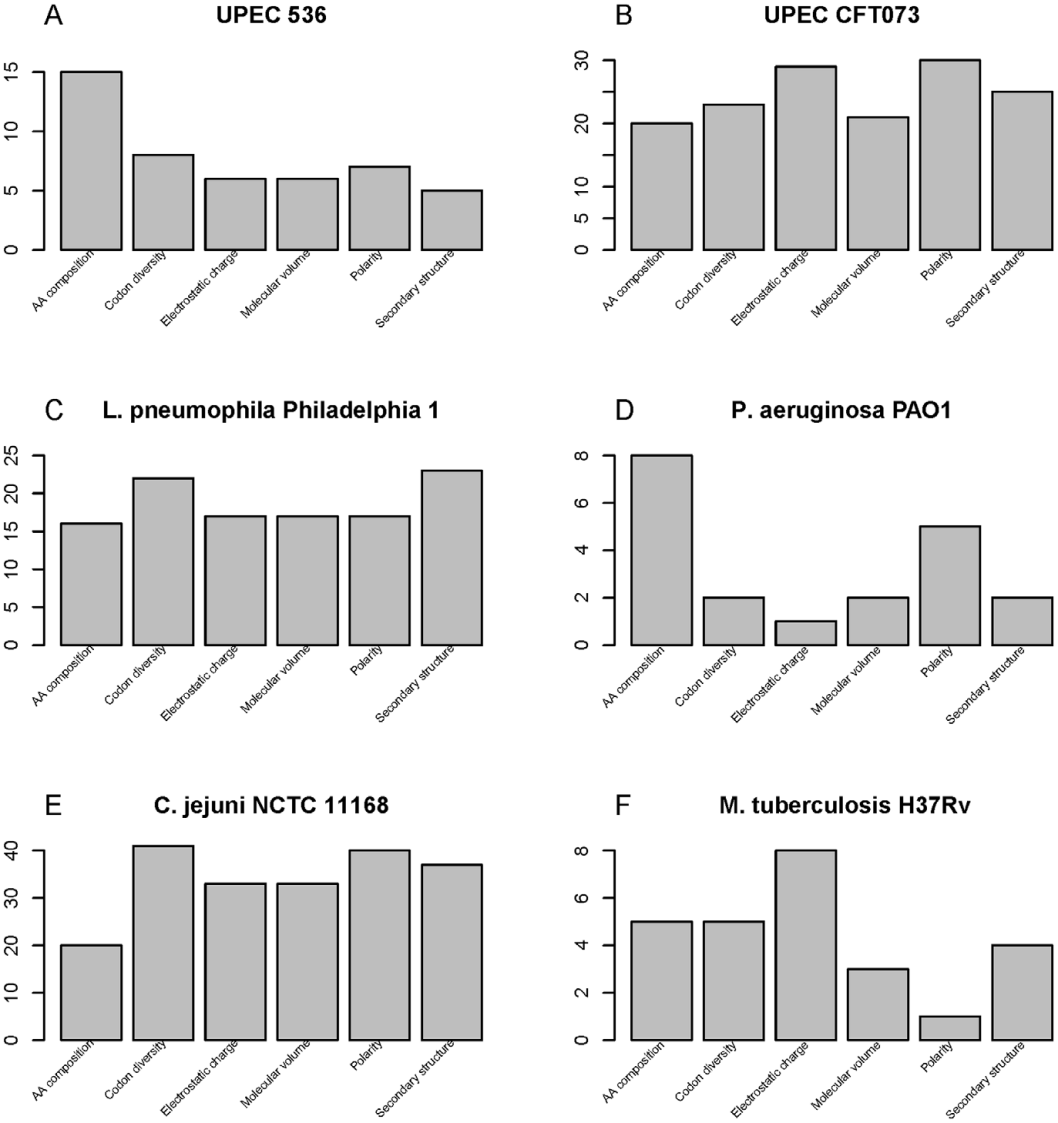
The distribution of the number of features of the optimal feature subset for each of the six species. In the feature space, all the features can be classified into six classes: amino acid composition, codon diversity, electrostatic charge, molecular volume, polarity and secondary structure.

**Figure 3:**
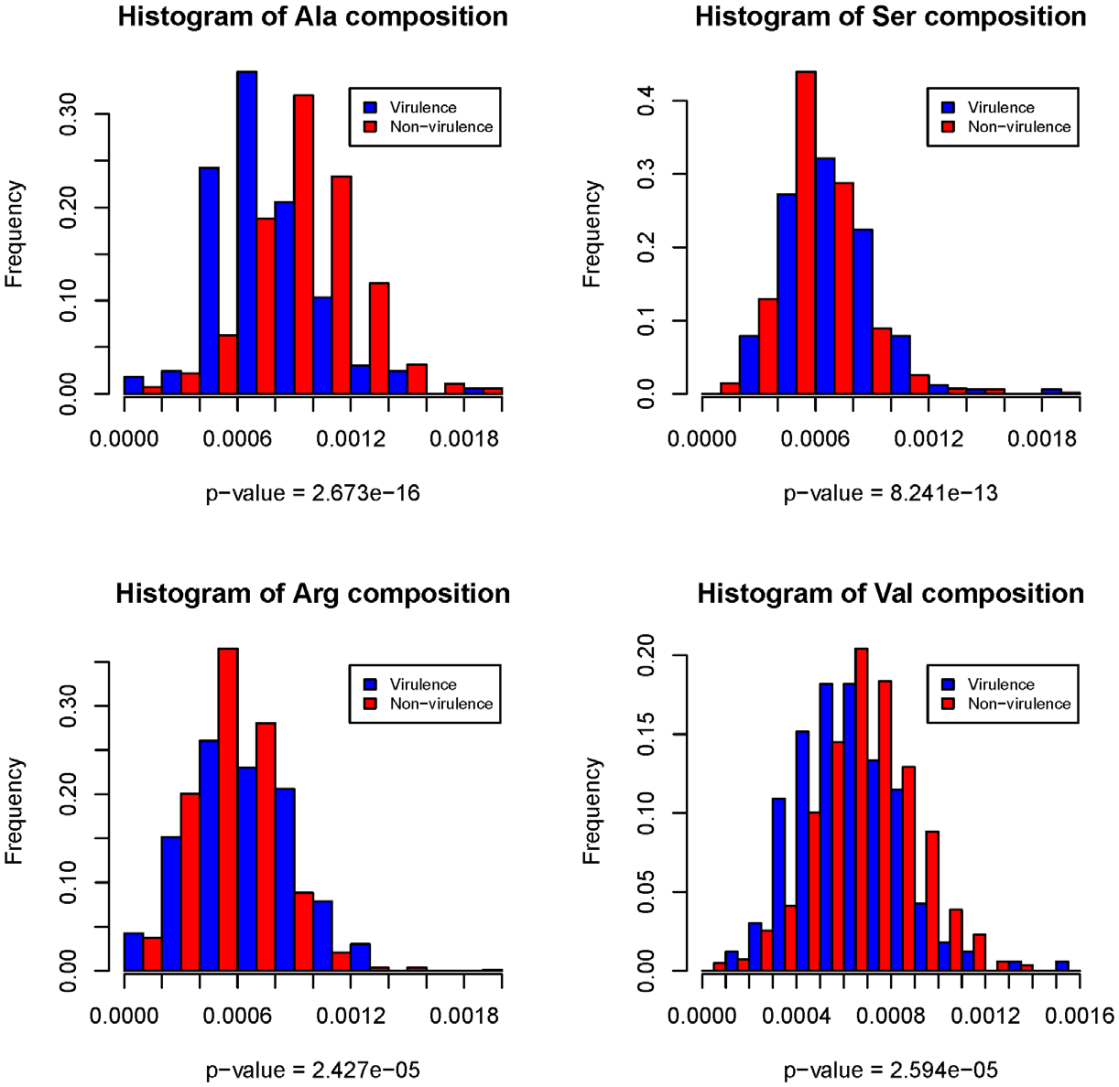
Histogram illustration to show the difference of the amino acid occurrence frequency between virulence and non-virulence factors. The histograms were plotted for Ala, Ser, Arg, and Val in UPEC 536, respectively. *X*-axis is the amino acid composition, while *y*-axis is the frequency of sequences that own the corresponding amino acid composition in the dataset. P-values are given by the Wilcoxon rank sum test and measure how much evidence we have against the null hypothesis that the amino acid composition distribution is the same for virulence and non-virulence factors. Traditionally, when p-value <0.05, we say the null hypothesis is rejected, that is, the amino acid composition distribution is significantly different for virulence and non-virulence factors. The feature distribution histograms and p-values show the difference of the amino acid composition frequencies between virulence and non-virulence factors is significant, and thus it is reasonable to pick out virulence factors from proteomes based on amino acid composition features.

### 6. Jackknife Cross-Validation and Evaluation

In statistical prediction, the jackknife cross-validation, also known as the leave-one-out cross-validation (LOOCV), is regarded as an objective and effective method to evaluate a classifier for its effectiveness in practical application. Accordingly, we adopted this method here to examine the quality of the present classifiers. During the jackknifing process, each of the proteins in the dataset was in turn singled out for testing by the classifier trained with the remaining proteins. To evaluate the performance quality, we calculated the following six indexes: sensitivity (*S_n_*), specificity (*S_p_*), precision (*P*), recall (*R*), accuracy (*AC*) and Matthews Correlation Coefficient (*MCC*), as formulated below:


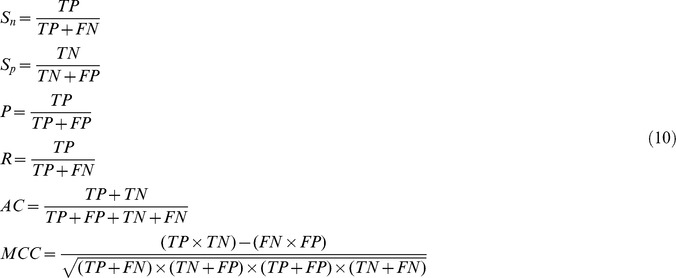


where *TP* represents the true positive, *TN* the true negative, *FP* the false positive, and *FN* the false negative. *S_n_*, *S_p_* and *AC* are the percentages of virulent proteins, non-virulent proteins and any proteins that are correctly predicted, respectively. Precision (*P*) is the proportion of the true positives against all the positive results (both true positives and false positives), while recall (*R*) in our classification context is referred to as the true positive rate (*S_n_*) in fact but is used in precision/recall curves. *MCC* equaling to 1 indicates a perfect prediction, whereas 0 means a completely random prediction. Then, we further calculated ROC score defined as the areas under the ROC curves, the plot of true positive rate (*S_n_*) as a function of the number of false positive rate (1–*S_p_*) by R package ROCR [28]. We also used ROCR to draw precision/recall curves for the comparison of the aforementioned three methods.

**Table 2:** Prediction based on feature selection method.

Species	*TP*	*FP*	*TN*	*FN*	*Sn*	*Sp*	*AC*	*MCC*
UPEC 536	87	96	732	78	0.52727	0.88406	0.82477	0.39489
UPEC CFT073	47	66	374	41	0.53409	0.85000	0.79735	0.34901
*L. pneumophila* Philadelphia 1	40	55	409	52	0.43478	0.88147	0.80755	0.31223
*P. aeruginosa* PAO1	49	100	656	102	0.32450	0.86772	0.77729	0.19326
*C. jejuni* NCTC 11168	36	36	356	42	0.46154	0.90816	0.83404	0.38189
*M. tuberculosis* H37Rv	26	41	291	40	0.39394	0.87651	0.79648	0.26882

**Table 3:** Prediction based on protein-protein interaction network.

Species	*TP*	*FP*	*TN*	*FN*	*Sn*	*Sp*	*AC*	*MCC*
UPEC 536	109	18	783	49	0.68987	0.97753	0.93014	0.73041
UPEC CFT073	80	19	359	3	0.96386	0.94974	0.95228	0.85480
*L. pneumophila* Philadelphia 1	59	20	397	30	0.66292	0.95204	0.90119	0.64503
*P. aeruginosa* PAO1	126	11	719	24	0.84000	0.98493	0.96023	0.85560
*C. jejuni* NCTC 11168	63	11	378	15	0.80769	0.97172	0.94433	0.79612
*M. tuberculosis* H37Rv	22	15	291	44	0.33333	0.95098	0.84140	0.36292

**Figure 4:**
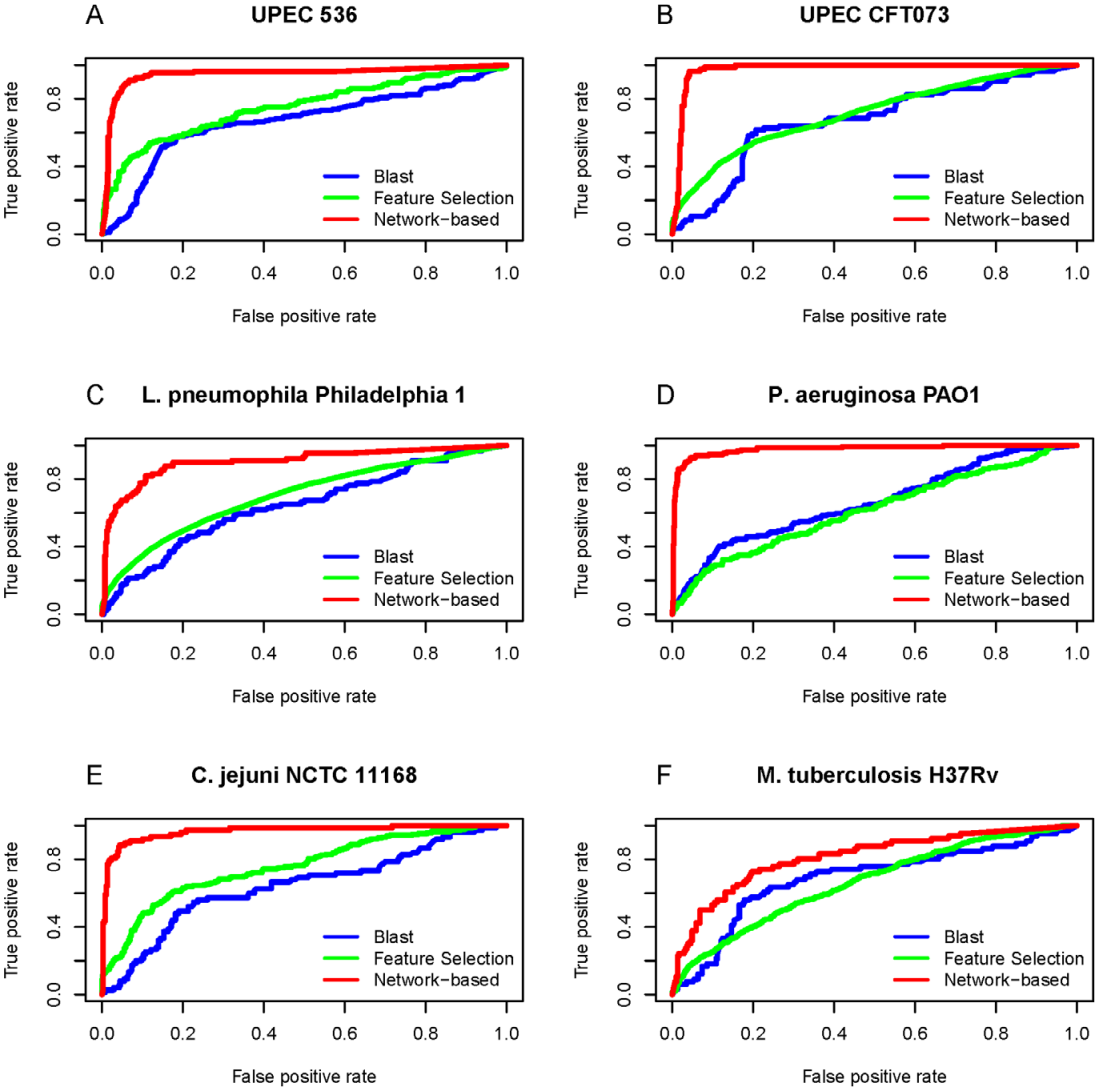
The ROC curves of true positive vs. false positive for the three different prediction methods. The curves for the network-based method are colored in red, while those for the BLAST method and the feature selected method in blue and green respectively. It can be seen that of the three methods, the network-based method had the best performance for all the following six cases: (A) UPEC 536; (B) UPEC CFT073; (C) *L. pneumophila* Philadelphia 1; (D) *P. aeruginosa* PAO1; (E) *C. jejuni* NCTC 11168; (F) *M. tuberculosis* H37Rv.

**Figure 5:**
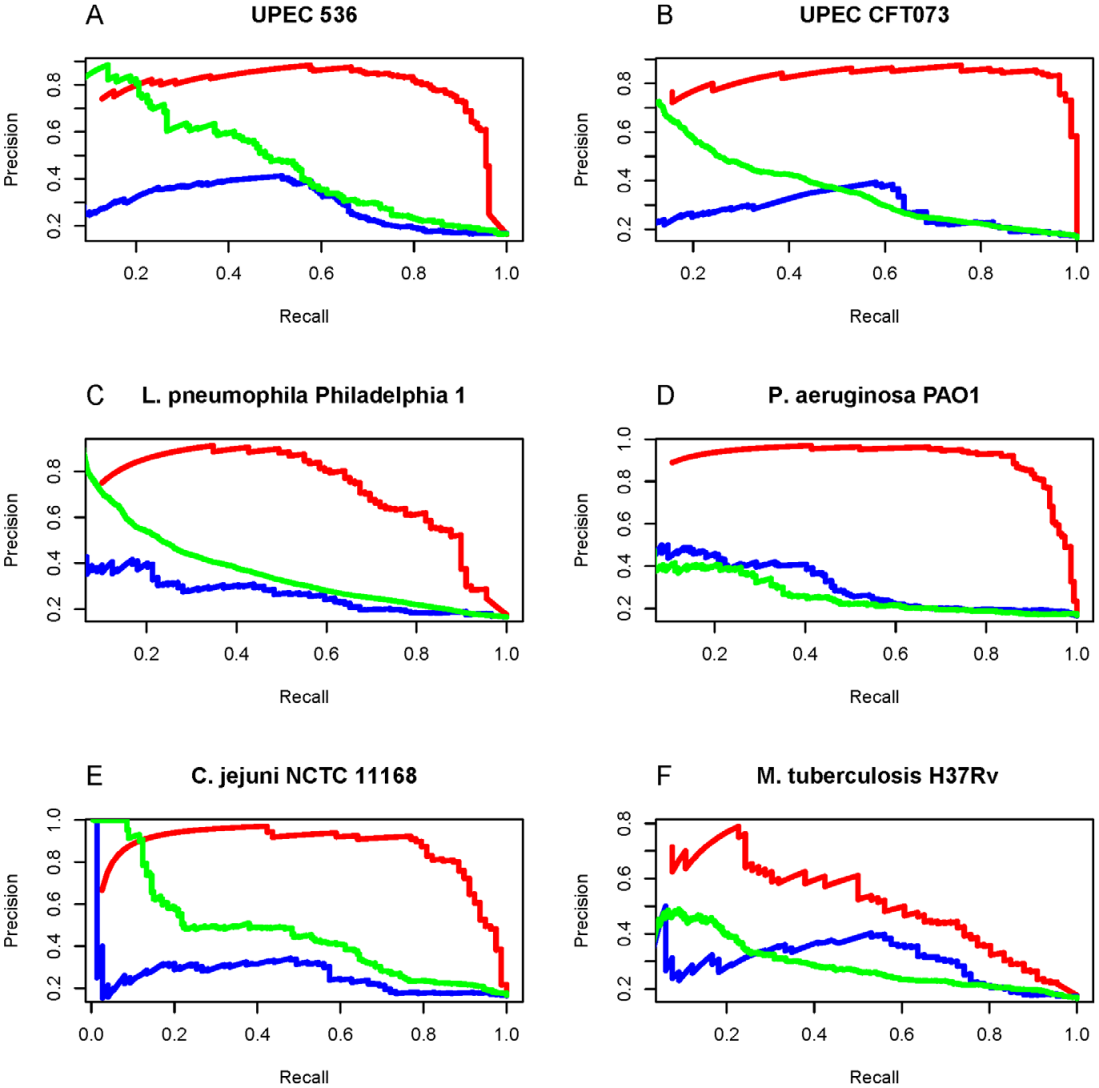
The Precision/recall curves for the three different prediction methods. See the legend of **Figure 4** for further explanation.

## Results and Discussion

### 1. Virulence Factors and Databases

By the use of the molecular version of Robert Koch’s postulates, which built a causal relationship between pathogens and disease, Stanley Falkow attempted to provide a definition of the term ‘virulence factor’: (1) the potential virulence factor gene should be found in all pathogenic strains of the genus or species but be absent from non-pathogenic strains; (2) virulence of the microbe with the inactivated gene should be less than that of the unaltered microbe in an appropriate animal model; (3) reintroduction of the relevant gene into the microbe should restore virulence in the animal model [29], [30]. His work has provided an experimentally rigorous approach to the study of virulence in certain bacterial pathogens. However, it should be noted that the definition of the virulence factor is also problematic and controversial [31], [32]. For example, some “classic” virulence factors, such as invasion genes (e.g., yjjp, ibeB and ompA), were also found in the genomes of commensal bacteria [32]. In spite of this imprecise definition, the virulence factor concept has still been used as a powerful engine in driving research in the fields of microbial pathogenesis and infectious diseases, and thus has greatly furthered our understanding of microbial pathogenesis [33]–[36].

Except VFDB and MvirDB mentioned above, several other databases have been developed specially for virulence factors, such as PHI-base (Pathogen Host Interations dataBase) [37], ARDB (Antibiotic Resistance Genes Database) [38] and ATDB (Animal Toxin Database) [39] and so on. Among these databases, VFDB was found to be the broadest and most comprehensive and had the highest quality with its curated dataset and virulence-guided classification system [5], [33]. Via exhaustive literature screening and expert review, VFDB has provided up-to-date information regarding experimentally validated bacterial virulence factors from genera of medically important bacterial pathogens. And therefore, we used the virulence factors from VFDB as our primary dataset.

**Table 4:** Comparison of several methods, including BLAST, Feature Selection, Network-based and VirulentPred, based on the testing dataset.

Method	*Sn*	*Sp*	*AC*	*MCC*
**UPEC 536**				
BLAST	0.54762	0.88718	0.82700	0.42332
Feature Selection	0.52381	0.86957	0.81125	0.37051
Network-based	0.78571	0.96500	0.93388	**0.76544**
VirulentPred	0.80952	0.69082	0.71084	0.38351
**UPEC CFT073**				
BLAST	0.66667	0.90476	0.86508	0.54233
Feature Selection	0.59091	0.87273	0.82576	0.42836
Network-based	0.90909	0.94898	0.94167	**0.81755**
VirulentPred	0.90909	0.62727	0.67424	0.40093
***L. pneumophila*** ** Philadelphia 1**				
BLAST	0.25000	0.81416	0.71533	0.06131
Feature Selection	0.54167	0.80172	0.75714	0.29611
Network-based	0.70833	0.96262	0.91603	**0.70743**
VirulentPred	0.95833	0.50000	0.57857	0.34982
***P. aeruginosa*** ** PAO1**				
BLAST	0.55263	0.86885	0.81448	0.39494
Feature Selection	0.34211	0.81482	0.73568	0.14347
Network-based	0.78947	0.95109	0.92342	**0.73300**
VirulentPred	0.84211	0.61376	0.65198	0.34134
***C. jejuni*** ** NCTC 11168**				
BLAST	0.50000	0.84211	0.78261	0.31437
Feature Selection	0.45000	0.86735	0.79661	0.30571
Network-based	0.75000	0.98980	0.94915	**0.81074**
VirulentPred	0.90000	0.41837	0.50000	0.24819
***M. tuberculosis*** ** H37Rv**				
BLAST	0.41176	0.82432	0.74725	0.22221
Feature Selection	0.29412	0.81928	0.73000	0.10649
Network-based	0.35294	0.95000	0.84536	0.37876
VirulentPred	0.76471	0.75904	0.76000	0.41840

**Figure 6:**
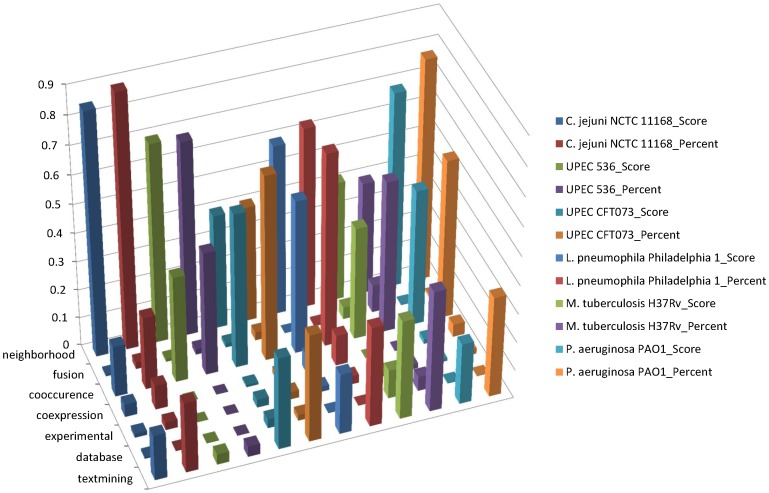
The functional associations of virulence factors in the STRING database. For each protein-protein interaction in the STRING database, there are seven evidence channels and each is assigned a confident subscore and then integrated to a combined score to show the possibility of the interaction. We analyzed all the interactions of virulence factors of six species, and computed the mean scores of seven evidence channels and percents of each evidence channel that had a score more than 0. After the normalization based on the combined score, we found that gene neighborhood and co-occurrence were the main associations between these virulence factors.

### 2. Results by BLAST

At first, we conducted the homology search for each species by BLASTP with the cutoff of HSP score being 90. However, most of the proteins (more than 80 percent) in the training dataset will be discarded for the poor homology among them. Thence, in the following study, to make use of the most of the data, no cutoff was set for the BLAST method. If the ratio of the smallest e-values of positive and negative samples was less than one, then the query protein was assigned to the virulence factor class regardless of how poor the alignment was; if not, non-virulence factor class. In some cases, it was also possible that no hit whatsoever existed for a query protein, and then the query protein would be excluded from the training dataset. For example, in UPEC 536, among 993 (207×6×0.8) proteins, 960 were predicted by the BLAST and 33 proteins were discarded. The prediction results are given in Table 1. As can be seen, the *S_n_*, *S_p_*, *AC*, and *MCC* for UPEC 536 were 0.460, 0.869, 0.800 and 0.315, respectively. The results of the other 5 species are also shown in Table 1. We can find that the overall prediction accuracies are around or less than 0.8.

**Figure 7:**
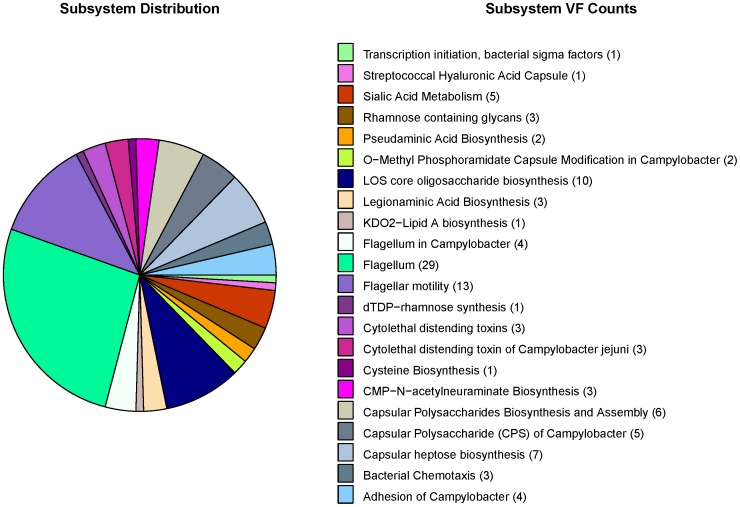
Enrichment of virulence factors in SEED subsystems by *C. jejuni* NCTC 11168. VF: virulence factor.

### 3. Results by the Feature Selection Method

We also applied feature selection method to predict whether a protein is a virulence factor or not. The model was constructed as follows. First of all, each of the proteins in the training dataset was coded as a 270-D feature vector in the feature space (see Section 4 of Materials and Methods). Then, the mRMR program was run to rank the 270 features according to the criteria of Maximum Relevance and Minimum Redundancy. The mRMR-ranked features can be found in Table S1 and will be participated in IFS procedure for feature selection and analysis. For each feature subset, a NNA classifier was built and its prediction accuracy was calculated by the jackknife cross-validation. Based on the number of the features in a feature subset and the corresponding prediction accuracy, we plotted the IFS curve (Figure 1). Again take UPEC 536 for example: it was observed that when the feature subset contained the first 47 features, the prediction accuracy got the highest value of 0.824773. Hence, the optimal prediction model should be constructed by the first 47 features in the mRMR feature list. For the other five species, the optimal number of features and the corresponding accuracy were (148; 0.797348), (112; 0.807554), (20; 0.777288), (204; 0.834043) and (26; 0.796482), respectively.

As described in the Materials and Methods, two kinds of features were used to code protein sequences. They were conventional amino acid compositions and pseudo-amino acid compositions, and the latter was based on 5 kinds of physicochemical and biochemical properties of amino acids, such as the codon diversity, electrostatic charge, molecular volume, polarity and secondary structure. The distribution of the number of features in each property in the optimized feature subset was investigated and shown in Figure 2. As the panel A of the figure showed that, in the optimized feature subset of UPEC 536, there were 15 features of amino acid compositions, 8 features of codon diversity, 6 features of electrostatic charge, 6 features of molecular volume, 7 features of polarity and 5 features of secondary structure. This indicated that both amino acid composition and pseudo-amino acid composition contributed to the prediction of virulence factors and that conventional amino acid composition may play an irreplaceable role in the prediction. Furthermore, the amino acid composition analysis of virulence and non-virulence factors revealed some interesting results. According to the criteria of maximum relevance to the target (Table S1), we selected the top 4 amino acid composition features ranked by mRMR to investigate the feature distribution between virulence and non-virulence factors (Figure 3). It was observed that compositions of residues Ala, Ser, Arg and Val, corresponding to AA composition1, AA composition16, AA composition15 and AA composition18 in the Table S1 respectively, contributed significantly to the classification for virulence and non-virulence factors. This was supported by the discovery of Aarti Garg et al.’s research [12]. Amino acid compositions had been successfully applied to the predictions of antimicrobial peptides [24], bacterial virulent proteins [12] and subcellular localization [40], [41], etc. And in many cases the approach outperformed the homology searching methods [12], [40], consistent with our results.

By analyzing the feature subset that achieved the best prediction accuracy for each species (Figure 2), it was revealed that the distribution of the features was different among the six species. For UPEC 536 and *P. aeruginosa* PAO1, conventional amino acid compositions played the most importance role, while for the other 4 species, pseudo-amino acid components such as codon diversity, electrostatic charge, polarity and secondary structure contributed more towards the prediction. The reasons may come from two factors. One is that the completeness of the annotation of virulence factors in each species is not the same: some may be studied by more research groups and has more detailed and accurate annotations. The other may be due to the inaccurate annotation where some virulence factors are still annotated as non-virulence factors.

Listed in Table 2 are the results obtained by the feature selection method on the six species via the jackknife tests.

### 4. Performance of the Network-Based Method

From STRING, the probabilistic confidence scores of interactions between proteins can usually be acquired, which can then be used to investigate biological problems [16], [42], [43]. However, some proteins may not interact with any of other proteins in the same training dataset. Take UPEC 536 as an example, only 959 proteins in its training dataset have interactions with other proteins, while the remaining 993−959 = 34 proteins have no interactions at all with the other proteins although they may interact with proteins outside training dataset. Considering the negative dataset was generated randomly, it is always possible that some proteins do not interact with any others in the training dataset. One feasible solution is to put all the non-virulence factors in STRING into the negative dataset. Unfortunately, this would make the size of the negative samples so large that *S_n_* would be very low, though *AC* could be high. In order to balance the positive and negative samples, we tested the performance by setting the ratio between positive samples and negative samples to be 1∶2, 1∶5 and 1∶10. And we found that when the ratio was 1∶5, we obtained the desirable performance. For the other five species (i.e., UPEC CFT073, *L. pneumophila* Philadelphia 1, *P. aeruginosa* PAO1, *C. jejuni* NCTC 11168 and *M. tuberculosis* H37Rv), the corresponding numbers of proteins without any interaction with the others are 528−461 = 67, 556−506 = 50, 907−880 = 27, 470−467 = 3 and 398−372 = 26, respectively. All these proteins were discarded.

Listed in Table 3 are the results obtained by the current network-based method on the six species via the jackknife tests. As we could see from the table, the *AC* values were more than 0.90 for all species except *M. tuberculosis* H37Rv, significantly higher than those by either the BLAST method or the feature selection method, indicating that the current network-based method is quite promising that may hold very high potential for identifying virulence factors in various organisms. However, it was noted that although the *AC* value achieved by the network-based method for *M. tuberculosis* H37Rv was higher than those by the BLAST and feature selection methods, the value was only 0.84140, much less than those for the other five species. The poor prediction performance for *M. tuberculosis H37Rv* might be due to that the quality of protein-protein interaction data for this organism in the STRING database is much poorer [44].

### 5. Comparison between Network-based and Other Methods

In this study, we developed three different methods to identity virulence factors. As shown from **Tables** **1**, **2**, and **3**, the network-based method significantly outperformed the BLAST method and feature selection method. Meanwhile, we also tried to perform the ROC and precision/recall comparisons. For the BLAST method, when the query protein sequence was very similar to some of the protein sequences in the database, the e-value would be close to zero, and hence their corresponding distance would also near zero in the feature selection method as described above. Consequently, many ratios would have extreme values, making the ROC and precision/recall curves for both BLAST and feature selection methods look abnormal. To tune this kind of extreme values, let us adopt the following monotone decreasing function


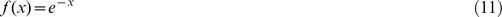


where *x* is either the e-value or distance. By means of Eq. 11, all the e-values and distances could be mapped into the region of (0,1]. After such a transformation, we redrew the ROC and precision/recall curves (Figures 4 and 5). As expected, the two kinds of curves have showed once again that the network-based method achieved the best performance among the three methods for all the six species.

Moreover, based on the independent testing dataset for the six species, we did plan to compare the prediction performance of our three methods with other existing methods, including VirulentPred [12] and Virulent-GO [13]. Unfortunately, no downloadable or online tool whatsoever was available for Virulent-GO. Thus, only the comparison with VirulentPred was made here as a compromise. The concrete comparison procedures are as follows. The positive and negative testing dataset was submitted onto the VirulentPred online service (http://203.92.44.117/virulent/submit.html) directly with default parameters. For our three methods, it should be noticed that the feature set used to code the testing dataset in the feature selection method was the optimal subset, which was obtained from the training dataset. The results of *Sn*, *Sp*, *AC* and *MCC* were also calculated for each method, respectively. As can be seen from Table 4, the network-based method achieved much better prediction performance than the BLAST and feature selection methods, too. Although the value of *Sn* by VirulentPred was slightly higher than that by the network-based method, the value of *Sp* by the former was much lower than that by the latter, indicating that the false positive outcome was really a serious problem for VirulentPred and hence leading to its poor prediction accuracy (*AC*) and *MCC*. As for *M. tuberculosis* H37Rv, Zhou and his colleagues [44] have demonstrated that the protein-protein interaction data of this organism in the STRING database is of low quality and thus may unfavorably affect our network-based method. Accordingly, it was not surprising that the performance on the testing dataset for such species was quite poor when compared with the other five. Taken together, we can draw a conclusion that the method based on the STRING networks is really better in identifying the bacterial virulence factors.

### 6. From the Sequence to the Network

Determining protein function is one of the most challenging problems in the post-genomic era. In this context, sequence-based methods such as BLAST are the primary tools to deal with this kind of problems. However, their accuracy is considerably affected by the type and amount of information on the specific protein family. Also, these methods would fail for those systems that contain a significant proportion of novel proteins without functionally known homologous counterparts in the current databases. Therefore, many new computational methods have been developed to infer the protein function using the principle of guilt-by-association of other functional properties to complement the sequence-based methods [45]. Our method based on the STRING protein-protein interaction network reflects one of the efforts in this regard. As the cornerstone in the current network-based method, the STRING database quantitatively integrates the interaction data from many information sources such as phylogenetic, experimental and existing knowledge information, extending the direct (physical) associations to the indirect (functional) associations. We have analyzed the detailed sub-score information of our STRING network data for the virulence factors in the six species. It was found that most of the interactions among virulence factors in the STRING database were functional associations, mainly with the neighborhood and co-occurrence associations (Figure 6). In view of this, we further studied the locations of the virulence factors in the genomes and biological processes they were involved in.

It has been noted by previous investigators [32], [46] that many virulence factors are presented in the pathogenicity islands involved in horizontal gene transfer. In 2009, with the number and diversity of bacterial genomes sequenced, a systematic large-scale analysis across diverse genera has indicated that virulence factors are disproportionately associated with genomic islands (GIs) [33]. Subsequently, we mapped our virulence factors of the six species to the SEED subsystems by the SEED Viewer version 2.0 (http://pubseed.theseed.org/seedviewer.cgi) [47]. In the microbial genome annotation, the SEED is the first annotation environment that curates genomic data via the curation of subsystems by an expert annotator across many genomes, not on a gene-by-gene basis. These subsystems group genes by the pathways or structures in which they participate. For instance, type 4 secretion and conjugative transfer are composed of a set of functional roles that some proteins perform (type IV secretion system protein VirD4, inner membrane protein forms channel for type IV secretion of T-DNA complex VirB3 and minor pilin of type IV secretion complex VirB5, etc.). Our results revealed that more than half of mapped virulence factors participated in a specific biological process or structural complex with at least one other virulence factor (Figure 7, Figures S1, S2, S3, S4, and S5). As Figure 7 showed, in *C. jejuni* NCTC 11168, as many as 29 and 13 virulence factors were involved in the flagellum subsystem and flagellar motility subsystem, respectively. Flagella belong to a major virulence factor in Campylobacter in VFDB, and can penetrate the mucus barrier and are important for intestinal colonization. Clusters of virulence factors in prokaryotic genomes and enrichments in biological pathways made it possible for their functional associations such as neighborhood and co-occurrence to be common and confident in the STRING database.

Our network-based method was based on hypothesis that proteins participating in the same cellular processes or being localized at the same cellular compartment usually share similar functions. This is reasonable because a pair of proteins participating in a same pathway or locating in a same complex is many folds more likely to interact with each other than a random pair of proteins [48]. In fact, during the course of infecting susceptible hosts, it is necessary for multiple virulence factors in bacterial pathogens to cooperate with each other [13], [34], [49]. For example, it has been shown that the prototypical type 1 secreted toxin, á-hemolysin (HlyA) is encoded by UPEC 536 and CFT073 and its expression is associated with increased clinical severity in the urinary tract infections patients [50]. However, the HlyA protein requires a post-translational modification for activity. The inactive protoxin pro-HlyA is activated by another virulence factor protein HlyC, which is an acyl carrier protein that acts as the fatty acid donor and is responsible for acylation of HylA, resulting in toxin activation [49]. Another example is that the secreted virulence factors by *Pseudomonas aeruginosa*, including â-lactamase, alkaline phosphatase, hemolytic phospholipase C, and Cif, are not released individually as naked proteins into the surrounding milieu. Instead, it is the bacterial-derived outer membrane vesicles (OMV) that deliver these virulence factors simultaneously and directly into the host airway epithelial cells in a coordinated manner [34]. In addition, Lilburn et al. [42] also proposed an approach by assembling a list of known virulent proteins and using these proteins as bait proteins in STRING functional association network to detect candidate virulent proteins involving in virulence in *Vibrio cholerae*, including proteins that are overlooked because of the incomplete annotation or the requirement of a follow-up investigation to confirm their roles in virulence. All these facts are consistent with the notion that virulent functions depend on the interaction of a large number of proteins. That is the essence of why the STRING network-based method is able to perform better than the sequence-based methods such as BLASTP and feature selection method (Tables 1, 2, 3, 4 and Figures 4 and 5).

### 7. Application and Improvement

Although the network-based method was merely tested for the proteins in six species, the high success rates obtained indicated the promising potential to be applied to other species as well. At present, we only considered virulence factors annotated in VFDB and protein-protein interactions in STRING database. Many other databases, such as MvirDB and SwissProt [51], also contain a large number of virulence factors, some of which are not collected in VFDB. Accordingly, for any other given bacterial species, we can also use the current network-based method to identify the virulence factors concerned once significant statistical data are available for the species. In other words, the current method can be easily extended to identify the virulence factors in many other bacterial species.

Despite quite high prediction accuracy by the network-based method, the following limitations should be pointed out. Firstly, some of the hypothetical non-virulent proteins in the training set could turn out to be virulence factors after more of their functions are determined in future. It will be less of a problem when more proteins are accurately annotated by experiments. Secondly, some protein-protein interactions from STRING database might not be reliable, such as the case in *M. tuberculosis* H37Rv. Also, some of the methods that generate protein interaction data – e.g., two-hybrids or gene neighbor – are susceptible to noise and might have a high false-positive rate [52]–[54]. Nevertheless, the STRING by combining the protein-protein interactions from multiple sources could improve their expected accuracy with at least 80% for more than half of the genes, clearly demonstrating the reliability of the data [55] in many cases. With enhanced quality of this small fraction of PPI networks in STRING, the performance of our network-based method can be further improved. Thirdly, the above network-based method has only taken into account of the neighbors that directly interact with the query protein, without considering the full topology of the network, during the prediction process. Yet it has been observed that, up to 69% of yeast proteins share functions with their indirect interaction partners, while only 48% share functions with their immediate interaction neighbors, as indicated in BioGrid [56]. Lastly, since the pathogenicity mechanism involves the interactions between the host and pathogen proteins [57], [58], more information about these kinds of interactions would be very useful in improving the methodology and even providing some clues or insights for revealing the mechanism.

## Supporting Information

Figure S1 **Enrichment of virulence factors in SEED subsystems by UPEC 536.** VF: virulence factor.
(TIFF)

Figure S2 **Enrichment of virulence factors in SEED subsystems by UPEC CFT073.** VF: virulence factor.
(TIFF)

Figure S3 **Enrichment of virulence factors in SEED subsystems by** ***L. pneumophila*** **Philadelphia 1.** VF: virulence factor.
(TIFF)

Figure S4 **Enrichment of virulence factors in SEED subsystems by** ***P. aeruginosa*** **PAO1.** VF: virulence factor.
(TIFF)

Figure S5 **Enrichment of virulence factors in SEED subsystems by** ***M. tuberculosis*** **H37Rv.** VF: virulence factor.
(TIFF)

Table S1 **The feature list for all the six species by mRMR.** The first part is the features ranked according to the criteria of maximum relevance to target. And the second part is the features ranked according to maximum relevance and minimum redundancy. The mRMR method could assign a score to each feature and then rank the features based on their scores. For a detailed description, see [25]. In the list of each part, the first column is the order of features ranked by mRMR; the second column is the original order of features input into mRMR; the third column is the names of features classified as amino acid composition, codon diversity, electrostatic charge, molecular volume, polarity and secondary structure; and the last column is the mRMR score.
(XLS)

## References

[1] Brzuszkiewicz E, Thurmer A, Schuldes J, Leimbach A, Liesegang H, et al (2011) Genome sequence analyses of two isolates from the recent Escherichia coli outbreak in Germany reveal the emergence of a new pathotype: Entero-Aggregative-Haemorrhagic Escherichia coli (EAHEC). Arch Microbiol 193: 883–891. 21713444 10.1007/s00203-011-0725-6 PMC3219860

[2] Korves T, Colosimo ME (2009) Controlled vocabularies for microbial virulence factors. Trends Microbiol 17: 279–285. 19577471 10.1016/j.tim.2009.04.002

[3] Wu HJ, Wang AH, Jennings MP (2008) Discovery of virulence factors of pathogenic bacteria. Curr Opin Chem Biol 12: 93–101. 18284925 10.1016/j.cbpa.2008.01.023

[4] Rasko DA, Sperandio V (2010) Anti-virulence strategies to combat bacteria-mediated disease. Nat Rev Drug Discov 9: 117–128. 20081869 10.1038/nrd3013

[5] Chen L, Xiong Z, Sun L, Yang J, Jin Q (2012) VFDB 2012 update: toward the genetic diversity and molecular evolution of bacterial virulence factors. Nucleic Acids Res 40: D641–645. 22067448 10.1093/nar/gkr989 PMC3245122

[6] Zhou CE, Smith J, Lam M, Zemla A, Dyer MD, et al (2007) MvirDB–a microbial database of protein toxins, virulence factors and antibiotic resistance genes for bio-defence applications. Nucleic Acids Res 35: D391–394. 17090593 10.1093/nar/gkl791 PMC1669772

[7] Gulig PA, de Crecy-Lagard V, Wright AC, Walts B, Telonis-Scott M, et al (2010) SOLiD sequencing of four Vibrio vulnificus genomes enables comparative genomic analysis and identification of candidate clade-specific virulence genes. BMC Genomics 11: 512. 20863407 10.1186/1471-2164-11-512 PMC3091676

[8] Wegmann U, O’Connell-Motherway M, Zomer A, Buist G, Shearman C, et al (2007) Complete genome sequence of the prototype lactic acid bacterium Lactococcus lactis subsp. cremoris MG1363. J Bacteriol 189: 3256–3270. 17307855 10.1128/JB.01768-06 PMC1855848

[9] Altschul SF (1997) Evaluating the statistical significance of multiple distinct local alignments. In: Suhai S, editor. Theoretical and Computational Methods in Genome Research. New York: Plenum. 1–14.

[10] Sachdeva G, Kumar K, Jain P, Ramachandran S (2005) SPAAN: a software program for prediction of adhesins and adhesin-like proteins using neural networks. Bioinformatics 21: 483–491. 15374866 10.1093/bioinformatics/bti028 PMC7109999

[11] Saha S, Raghava GPS (2006) VICMpred: An SVM-based Method for the Prediction of Functional Proteins of Gram-negative Bacteria Using Amino Acid Patterns and Composition. Genomics, Proteomics & Bioinformatics 4: 42–47. 10.1016/S1672-0229(06)60015-6 PMC5054027 16689701

[12] Garg A, Gupta D (2008) VirulentPred: a SVM based prediction method for virulent proteins in bacterial pathogens. BMC Bioinformatics 9: 62. 18226234 10.1186/1471-2105-9-62 PMC2254373

[13] Tsai C-T, Huang W-L, Ho S-J, Shu L-S, Ho S-Y (2009) Virulent-GO: Prediction of Virulent Proteins in Bacterial Pathogens Utilizing Gene Ontology Terms. International Journal of Biological and Life Sciences 5: 80–87.

[14] Ashburner M, Ball CA, Blake JA, Botstein D, Butler H, et al (2000) Gene ontology: tool for the unification of biology. Nature Genetics 25: 25–29. 10802651 10.1038/75556 PMC3037419

[15] Szklarczyk D, Franceschini A, Kuhn M, Simonovic M, Roth A, et al (2011) The STRING database in 2011: functional interaction networks of proteins, globally integrated and scored. Nucleic Acids Res 39: D561–568. 21045058 10.1093/nar/gkq973 PMC3013807

[16] Hu L, Huang T, Shi X, Lu WC, Cai YD, et al (2011) Predicting functions of proteins in mouse based on weighted protein-protein interaction network and protein hybrid properties PLoS ONE. 6: e14556. 10.1371/journal.pone.0014556 PMC3023709 21283518

[17] Chou KC (2001) Prediction of protein cellular attributes using pseudo amino acid composition. PROTEINS: Structure, Function, and Genetics (Erratum: ibid, 2001, Vol44, 60) 43: 246–255. 10.1002/prot.1035 11288174

[18] Chou KC (2005) Using amphiphilic pseudo amino acid composition to predict enzyme subfamily classes. Bioinformatics 21: 10–19. 15308540 10.1093/bioinformatics/bth466

[19] Lin H, Wang H, Ding H, Chen Y-L, Li Q-Z (2009) Prediction of Subcellular Localization of Apoptosis Protein Using Chou’s Pseudo Amino Acid Composition. Acta Biotheoretica 57: 321–330. 19169652 10.1007/s10441-008-9067-4

[20] Sahu SS, Panda G (2010) A novel feature representation method based on Chou’s pseudo amino acid composition for protein structural class prediction. Computational Biology and Chemistry 34: 320–327. 21106461 10.1016/j.compbiolchem.2010.09.002

[21] Fang Y, Guo Y, Feng Y, Li M (2008) Predicting DNA-binding proteins: approached from Chou’s pseudo amino acid composition and other specific sequence features. Amino Acids 34: 103–109. 17624492 10.1007/s00726-007-0568-2

[22] Atchley WR, Zhao J, Fernandes AD, Druke T (2005) Solving the protein sequence metric problem. Proc Natl Acad Sci U S A 102: 6395–6400. 15851683 10.1073/pnas.0408677102 PMC1088356

[23] Rubinstein ND, Mayrose I, Pupko T (2009) A machine-learning approach for predicting B-cell epitopes. Mol Immunol 46: 840–847. 18947876 10.1016/j.molimm.2008.09.009

[24] Wang P, Hu L, Liu G, Jiang N, Chen X, et al (2011) Prediction of antimicrobial peptides based on sequence alignment and feature selection methods. PLoS ONE 6: e18476. 21533231 10.1371/journal.pone.0018476 PMC3076375

[25] Peng H, Long F, Ding C (2005) Feature selection based on mutual information: criteria of max-dependency, max-relevance, and min-redundancy. IEEE Trans Pattern Anal Mach Intell 27: 1226–1238. 16119262 10.1109/TPAMI.2005.159

[26] Kohavi R, John GH (1997) Wrappers for feature subset selection. Artif Intell 97: 273–324.

[27] Friedman JH, Baskett F, Shustek LJ (1975) An algorithm for finding nearest neighbors. IEEE Transaction on Information Theory C-24: 1000–1006.

[28] Sing T, Sander O, Beerenwinkel N, Lengauer T (2005) ROCR: visualizing classifier performance in R. Bioinformatics. 21: 3940–3941. 10.1093/bioinformatics/bti623 16096348

[29] Falkow S (1988) Molecular Koch’s postulates applied to microbial pathogenicity. Rev Infect Dis 10 Suppl 2S274–276. 3055197 10.1093/cid/10.supplement_2.s274

[30] Falkow S (2004) Molecular Koch’s postulates applied to bacterial pathogenicity–a personal recollection 15 years later. Nat Rev Microbiol 2: 67–72. 15035010 10.1038/nrmicro799

[31] Casadevall A, Pirofski LA (1999) Host-pathogen interactions: redefining the basic concepts of virulence and pathogenicity. Infect Immun 67: 3703–3713. 10417127 10.1128/iai.67.8.3703-3713.1999 PMC96643

[32] Pallen MJ, Wren BW (2007) Bacterial pathogenomics. Nature 449: 835–842. 17943120 10.1038/nature06248

[33] Ho Sui SJ, Fedynak A, Hsiao WW, Langille MG, Brinkman FS (2009) The association of virulence factors with genomic islands. PLoS ONE 4: e8094. 19956607 10.1371/journal.pone.0008094 PMC2779486

[34] Bomberger JM, Maceachran DP, Coutermarsh BA, Ye S, O’Toole GA, et al (2009) Long-distance delivery of bacterial virulence factors by Pseudomonas aeruginosa outer membrane vesicles. PLoS Pathog 5: e1000382. 19360133 10.1371/journal.ppat.1000382 PMC2661024

[35] Isberg RR, O’Connor TJ, Heidtman M (2009) The Legionella pneumophila replication vacuole: making a cosy niche inside host cells. Nat Rev Microbiol 7: 13–24. 19011659 10.1038/nrmicro1967 PMC2631402

[36] Mellmann A, Harmsen D, Cummings CA, Zentz EB, Leopold SR, et al (2011) Prospective genomic characterization of the German enterohemorrhagic Escherichia coli O104: H4 outbreak by rapid next generation sequencing technology. PLoS ONE 6: e22751. 21799941 10.1371/journal.pone.0022751 PMC3140518

[37] Winnenburg R, Urban M, Beacham A, Baldwin TK, Holland S, et al (2008) PHI-base update: additions to the pathogen host interaction database. Nucleic Acids Res 36: D572–576. 17942425 10.1093/nar/gkm858 PMC2238852

[38] Liu B, Pop M (2009) ARDB–Antibiotic Resistance Genes Database. Nucleic Acids Res 37: D443–447. 18832362 10.1093/nar/gkn656 PMC2686595

[39] He QY, He QZ, Deng XC, Yao L, Meng E, et al (2008) ATDB: a uni-database platform for animal toxins. Nucleic Acids Res 36: D293–297. 17933766 10.1093/nar/gkm832 PMC2238984

[40] Garg A, Bhasin M, Raghava GP (2005) Support vector machine-based method for subcellular localization of human proteins using amino acid compositions, their order, and similarity search. Journal of Biological Chemistry 280: 14427–14432. 15647269 10.1074/jbc.M411789200

[41] Zhou GP, Doctor K (2003) Subcellular location prediction of apoptosis proteins. Proteins: Structure, Function, and Genetics 50: 44–48. 10.1002/prot.10251 12471598

[42] Gu J, Wang Y, Lilburn T (2009) A comparative genomics, network-based approach to understanding virulence in Vibrio cholerae. J Bacteriol 191: 6262–6272. 19666715 10.1128/JB.00475-09 PMC2753031

[43] Brouwers L, Iskar M, Zeller G, van Noort V, Bork P (2011) Network Neighbors of Drug Targets Contribute to Drug Side-Effect Similarity. PLoS ONE 6: e22187. 21765950 10.1371/journal.pone.0022187 PMC3135612

[44] Zhou H, Wong L (2011) Comparative analysis and assessment of M. tuberculosis H37Rv protein-protein interaction datasets. BMC Genomics 12 Suppl 3S20. 22369691 10.1186/1471-2164-12-S3-S20 PMC3333180

[45] Hawkins T, Kihara D (2007) Function prediction of uncharacterized proteins. J Bioinform Comput Biol 5: 1–30. 17477489 10.1142/s0219720007002503

[46] Croxen MA, Finlay BB (2010) Molecular mechanisms of Escherichia coli pathogenicity. Nat Rev Microbiol 8: 26–38. 19966814 10.1038/nrmicro2265

[47] Overbeek R, Begley T, Butler RM, Choudhuri JV, Chuang H-Y, et al (2005) The Subsystems Approach to Genome Annotation and its Use in the Project to Annotate 1000 Genomes. Nucleic Acids Research 33: 5691–5702. 16214803 10.1093/nar/gki866 PMC1251668

[48] Liu G, Li J, Wong L (2008) Assessing and predicting protein interactions using both local and global network topological metrics. Genome Inform 21: 138–149. 19425154

[49] Stanley P, Koronakis V, Hughes C (1998) Acylation of Escherichia coli hemolysin: a unique protein lipidation mechanism underlying toxin function. Microbiol Mol Biol Rev 62: 309–333. 9618444 10.1128/mmbr.62.2.309-333.1998 PMC98917

[50] Wiles TJ, Kulesus RR, Mulvey MA (2008) Origins and virulence mechanisms of uropathogenic Escherichia coli. Exp Mol Pathol 85: 11–19. 18482721 10.1016/j.yexmp.2008.03.007 PMC2595135

[51] The UC (2011) Ongoing and future developments at the Universal Protein Resource. Nucleic Acids Research 39: D214–D219. 21051339 10.1093/nar/gkq1020 PMC3013648

[52] von Mering C, Krause R, Snel B, Cornell M, Oliver SG, et al (2002) Comparative assessment of large-scale data sets of protein-protein interactions. Nature 417: 399–403. 12000970 10.1038/nature750

[53] Braun P, Tasan M, Dreze M, Barrios-Rodiles M, Lemmens I, et al (2009) An experimentally derived confidence score for binary protein-protein interactions. Nat Methods 6: 91–97. 19060903 10.1038/nmeth.1281 PMC2976677

[54] Shoemaker BA, Panchenko AR (2007) Deciphering protein-protein interactions. Part II. Computational methods to predict protein and domain interaction partners. PLoS Comput Biol 3: e43. 17465672 10.1371/journal.pcbi.0030043 PMC1857810

[55] von Mering C, Huynen M, Jaeggi D, Schmidt S, Bork P, et al (2003) STRING: a database of predicted functional associations between proteins. Nucleic Acids Res 31: 258–261. 12519996 10.1093/nar/gkg034 PMC165481

[56] Chua HN, Sung W-K, Wong L (2006) Exploiting indirect neighbours and topological weight to predict protein function from protein protein interactions. Bioinformatics 22: 1623–1630. 16632496 10.1093/bioinformatics/btl145

[57] Dyer MD, Neff C, Dufford M, Rivera CG, Shattuck D, et al (2010) The human-bacterial pathogen protein interaction networks of Bacillus anthracis, Francisella tularensis, and Yersinia pestis. PLoS ONE 5: e12089. 20711500 10.1371/journal.pone.0012089 PMC2918508

[58] Hogan DA, Kolter R (2002) Pseudomonas-Candida Interactions: An Ecological Role for Virulence Factors. Science 296: 2229–2232. 12077418 10.1126/science.1070784

