# Supplementary figures and images for: A Comparison of Computational Methods for Identifying Virulence Factors
Source: PLoS One. 2012 Aug 3;7(8):e42517. doi: 10.1371/journal.pone.0042517 (PMC3411817; doi:10.1371/journal.pone.0042517)

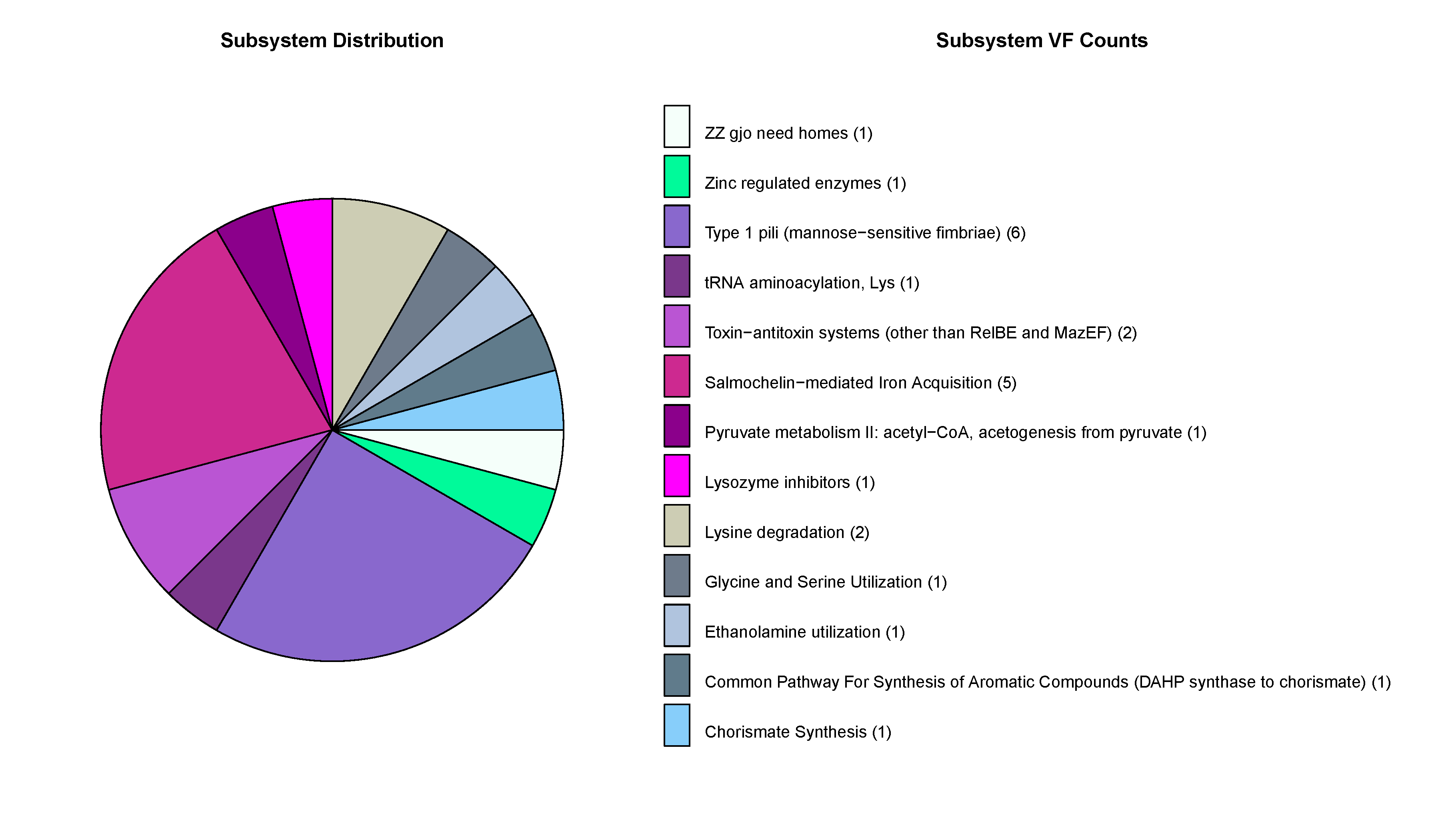

Supplement: Figure S1 — Enrichment of virulence factors in SEED subsystems by UPEC 536. VF: virulence factor. (TIFF) [file pone.0042517.s001.tiff]

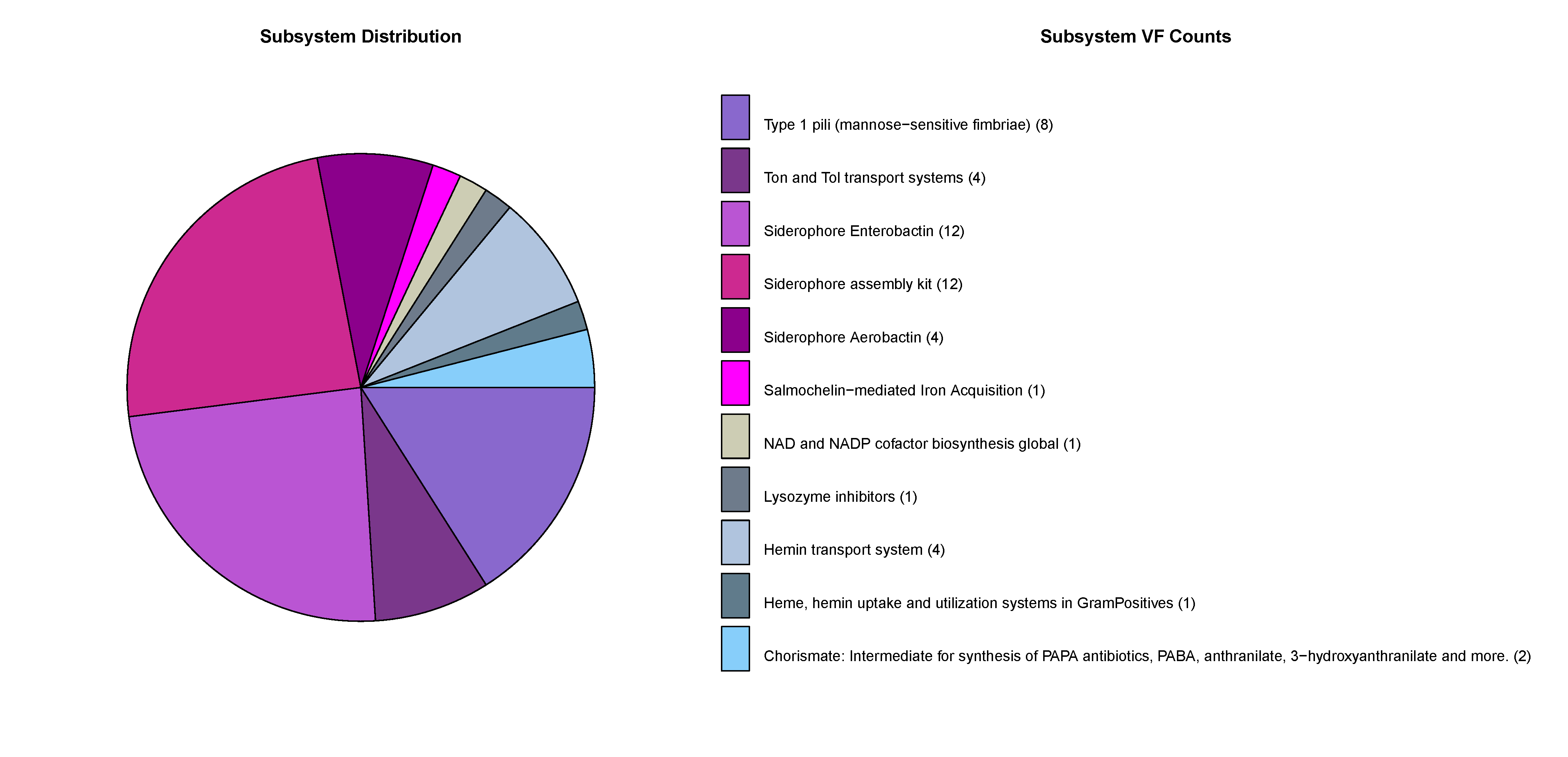

Supplement: Figure S2 — Enrichment of virulence factors in SEED subsystems by UPEC CFT073. VF: virulence factor. (TIFF) [file pone.0042517.s002.tiff]

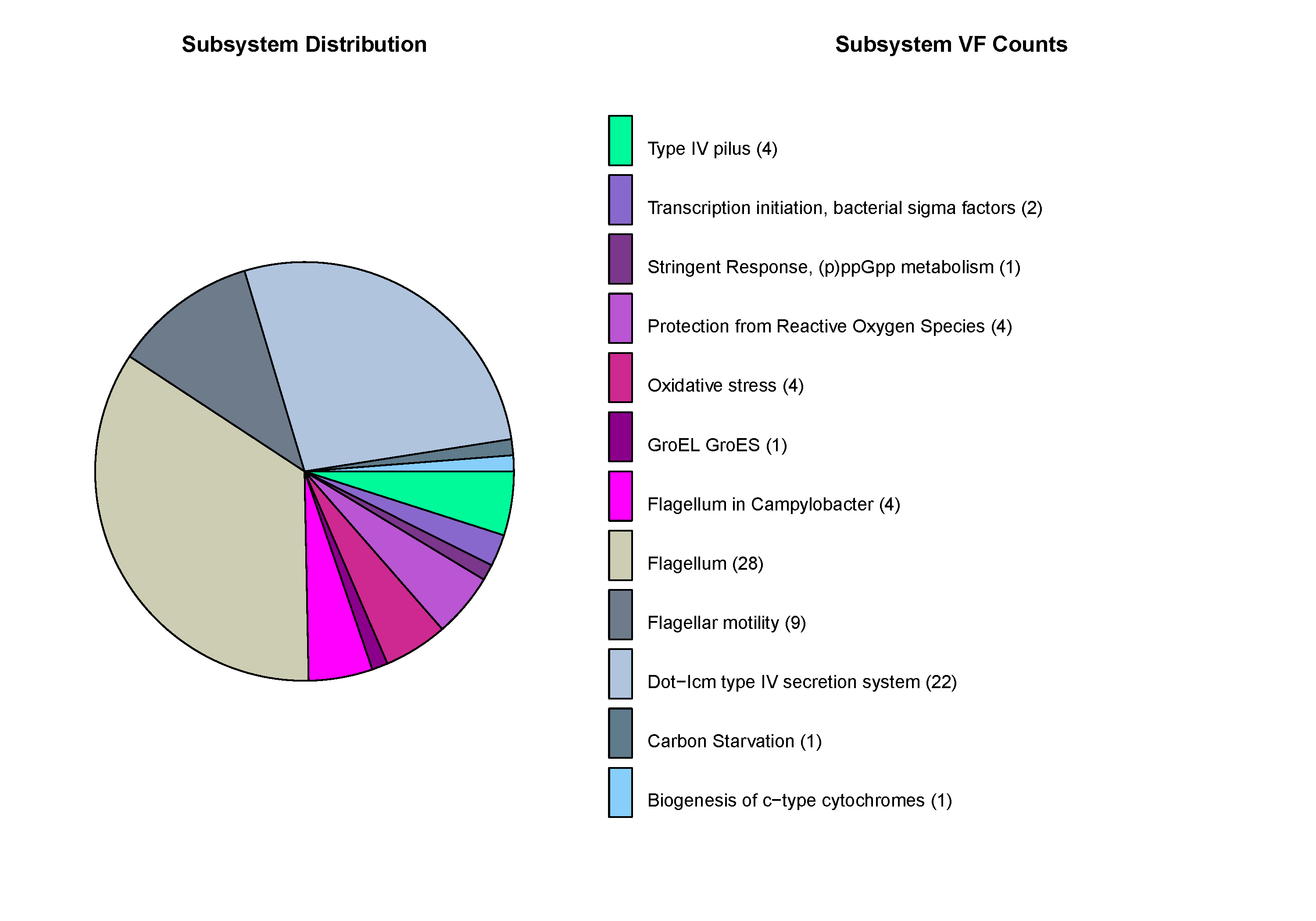

Supplement: Figure S3 — Enrichment of virulence factors in SEED subsystems by L. pneumophila Philadelphia 1. VF: virulence factor. (TIFF) [file pone.0042517.s003.tiff]

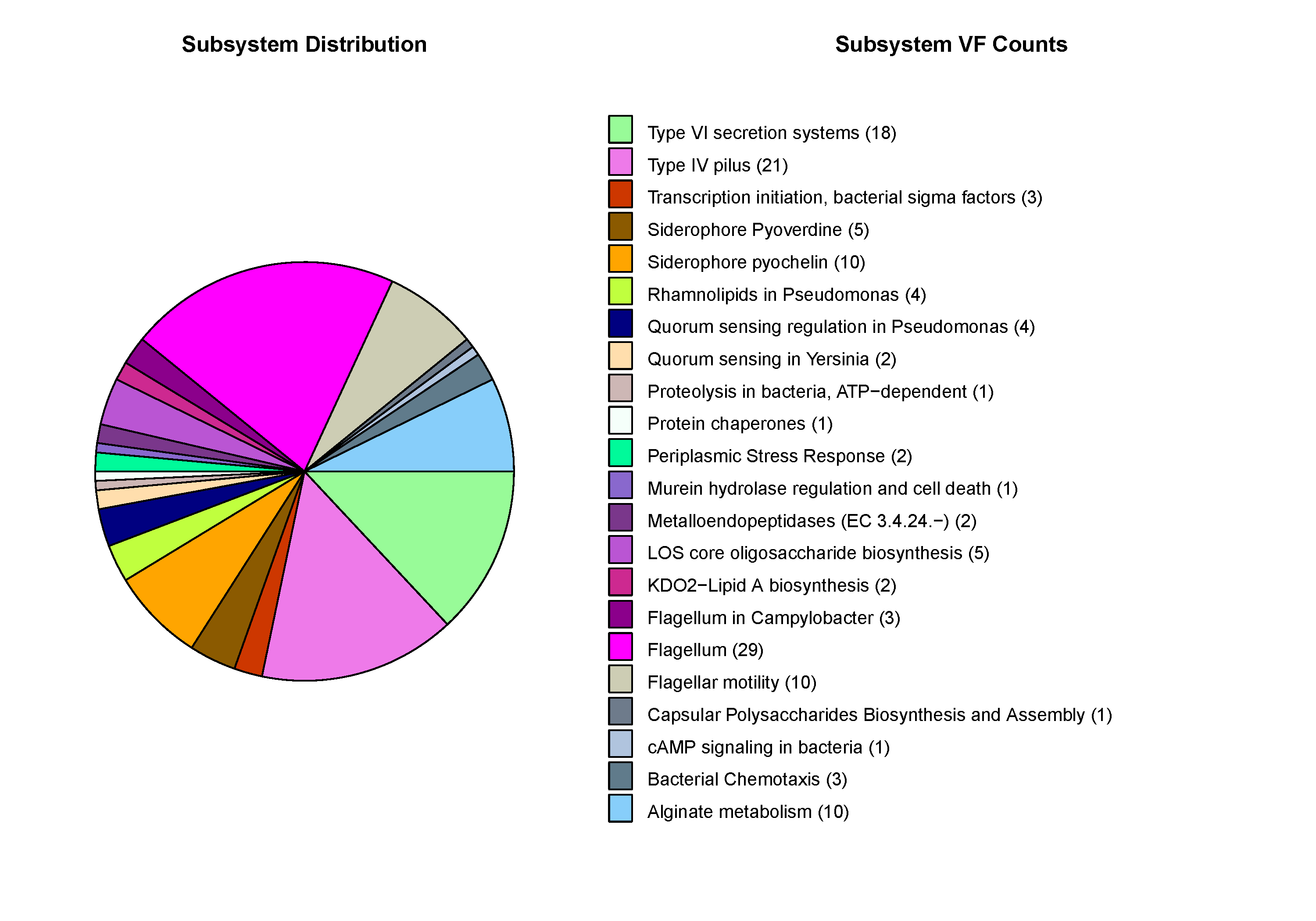

Supplement: Figure S4 — Enrichment of virulence factors in SEED subsystems by P. aeruginosa PAO1. VF: virulence factor. (TIFF) [file pone.0042517.s004.tiff]

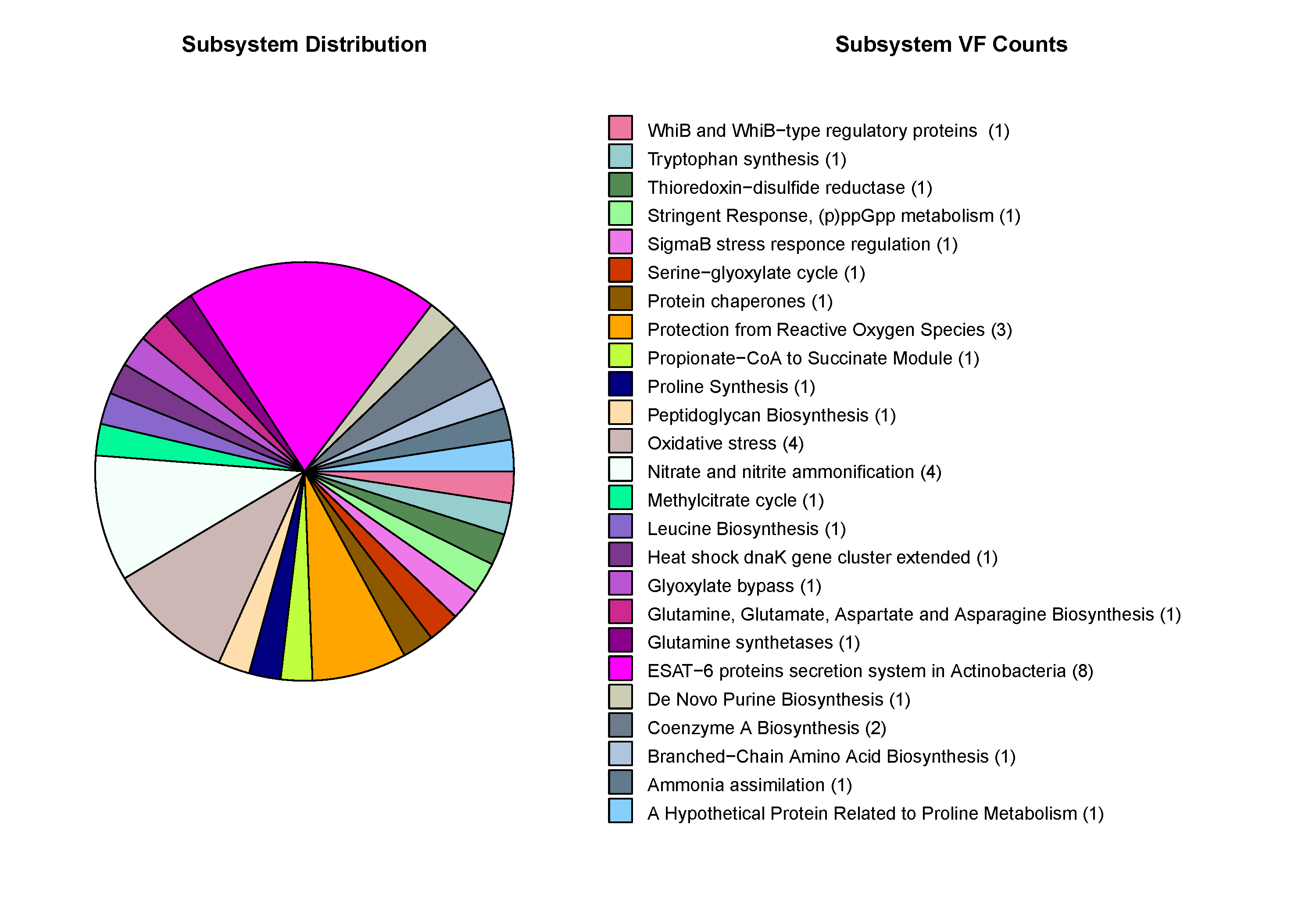

Supplement: Figure S5 — Enrichment of virulence factors in SEED subsystems by M. tuberculosis H37Rv. VF: virulence factor. (TIFF) [file pone.0042517.s005.tiff]
